# Suture button versus syndesmotic screw in ankle fractures - evaluation with 3D imaging-based measurements

**DOI:** 10.1186/s12891-021-04834-0

**Published:** 2021-11-22

**Authors:** Robert Hennings, Firas Souleiman, Martin Heilemann, Mareike Hennings, Alexis Klengel, Georg Osterhoff, Pierre Hepp, Annette B. Ahrberg

**Affiliations:** 1 Department of Orthopaedics, Traumatology and Plastic Surgery, University of Leipzig, Liebigstr. 20, 04103 Leipzig, Saxony Germany; 2 ZESBO - Centre for Research on Musculoskeletal Systems, University of Leipzig, Semmelweisstraße 14, 04103 Leipzig, Saxony Germany; 3 Department of Radiology, University of Leipzig, Liebigstr. 20, 04103 Leipzig, Saxony Germany

**Keywords:** Syndesmosis, Tibio-fibular, Syndesmotic screw, Suture button, 3D imaging, 3D measurement

## Abstract

**Background:**

Inadequate reduction of syndesmotic injuries can result in disabling clinical outcomes. The aim of the study was to compare syndesmosis congruity after fixation by syndesmotic screws (SYS) or a suture button system (SBS) using three-dimensional (3D) computed imaging techniques.

**Methods:**

In a retrospective single-center study, patients with unilateral stabilization of an ankle fracture with a syndesmotic injury and post-operative bilateral CT scans were analyzed using a recently established 3D method. The side-to-side differences were compared for tibio-fibular clear space (∆CS), translation angle (∆α), and vertical offset (∆z) among patients stabilized with syndesmotic screws or suture button system. Syndesmotic malreduction was defined for ∆CS > 2 mm and for |∆α| > 5°. ∆CS and ∆α were correlated with two-dimensional (2D) measurements.

**Results:**

Eighteen patients stabilized with a syndesmosis screw and 29 stabilized with a suture button system were analyzed. After stabilization, both groups revealed mild diastasis (SYS: mean ∆CS 0.3 mm, SD 1.1 mm vs SBS: mean ∆CS 0.2 mm, SD 1.2 mm, *p* = 0.710). In addition, both stabilization methods showed slight dorsalization of the fibula (SYS: mean ∆α 0.5°, SD 4.6° vs SBS: mean ∆α 2.1°, SD 3.7°, *p* = 0.192). Also, restoration of the fibula-to-tibia length ratio also did not differ between the two groups (SYS: mean Δz of 0.5 mm, SD 2.4 mm vs SBS: mean Δz of 0 mm, SD 1.2 mm; *p* = 0.477). Malreduction according to high ∆α was most common (26% of cases), with equal distribution between the groups (*p* = 0.234). ∆CS and ∆α showed good correlation with 2D measurements (*ρ* = 0.567; *ρ* = 0.671).

**Conclusion:**

This in vivo analysis of post-operative 3D models showed no differences in immediate post-operative alignment after syndesmotic screws or suture button system. Special attention should be paid to syndesmotic malreduction in the sagittal orientation of the fibula in relation to the tibia in radiological control of the syndesmotic congruity as well as intra-operatively.

## Background

There is broad agreement on the need for stabilization of an unstable distal tibio-fibular joint due to a syndesmotic injury in ankle fractures [1–3]. An unstable syndesmosis with diastasis results in eccentric stress on the tibio-talar joint and is associated with poor clinical outcome and higher rates of secondary osteoarthritis [2, 4–7]. Pre-operative evaluation of the syndesmosis using conventional radiographic imaging is difficult [8]. Therefore, intra-operative testing for instability is recommended [1]. If instability of the distal tibio-fibular joint is present, stabilization is indicated after anatomical reduction [1, 7]. The syndesmotic screw (SYS) and suture-button systems (SBS) are available for this purpose. In conventional radiographs, syndesmotic malreduction after stabilization is described in about 20% of the cases with a significantly worse functional outcome [9]. Using computed tomography (CT), however, syndesmotic malreduction rates of up to 52% have been reported [9, 10]. Therefore, because of the large interindividual and small intraindividual anatomic differences of the distal tibio-fibular joint, post-operative bilateral CT is recommended if no intra-operative CT control was performed [11–14]. Furthermore, two-dimensional (2D) measurements cannot fully describe the three-dimensional (3D) relationships of the syndesmosis [15, 16]. Meta-analyses have shown that stabilization with SBS may be less frequently associated with syndesmotic malreduction [17–19]. There is still debate about which of the two stabilization techniques should be preferred [20–23]. Therefore, in addition to the 2D assessment of fractures, 3D imaging is gaining importance for preoperative planning and the assessment of surgical outcomes [9, 15, 16, 24, 25]. The purpose of this study was to assess the post-operative alignment of syndesmosis fixation performed with either SYS or SBS using a 3D-based method [14].

## Methods

We performed a retrospective case control study at a level 1 trauma center. This study was conducted following approval from the local ethics committee (AZ 131/18-ek; AZ 361/19-ek) and was performed in accordance with the principles of the Declaration of Helsinki. Informed consent to participate in the study was given by all patients. Consecutive patients who underwent stabilization of the syndesmosis as part of surgical treatment of ankle fractures between 01/2008 and 12/2017 and met the inclusion criteria were included (Table 1). Fractures were classified according to the AO classification [26].

**Table 1:** Inclusion and exclusion criteria

Inclusion criteria	Exclusion criteria
• unilateral stabilization of the syndesmosis with SBS or SYS • anatomical reduction of the fractures • uninjured ankle without pathologies • postoperative CT included at least 10 cm proximal to the distal tibial plateau	• age < 18 years • Bilateral ankle and/or syndesmosis lesion • Pathologies of the uninjured ankle • Inadequate fracture reduction with bone steps > 2 mm. • Missing bilateral CT control • CT less than 10 cm proximal to the distal tibial plateau

### Operative management

All patients were treated according to the recommendations of the AO after written informed consent [27]. Following anatomical fracture reduction, osteosynthesis and verification of an instability under fluoroscopy, stabilization with visualization of the syndesmosis was performed with either a syndesmotic screw (SYS-group, 3.5 mm, DePuy-Synthes, West Chester, PA) or a suture-button device (SBS group, TightRope®, Arthrex, Naples, FL, USA) as preferred by the surgeon [27, 28]. The syndesmosis was opened as far as it was accessible through the lateral approach. Standard fluoroscopy (lateral and mortise view) was applied intra-operatively to control the reduction.

### Imaging, 3D measurements and outcome parameters

**Fig. 1:**
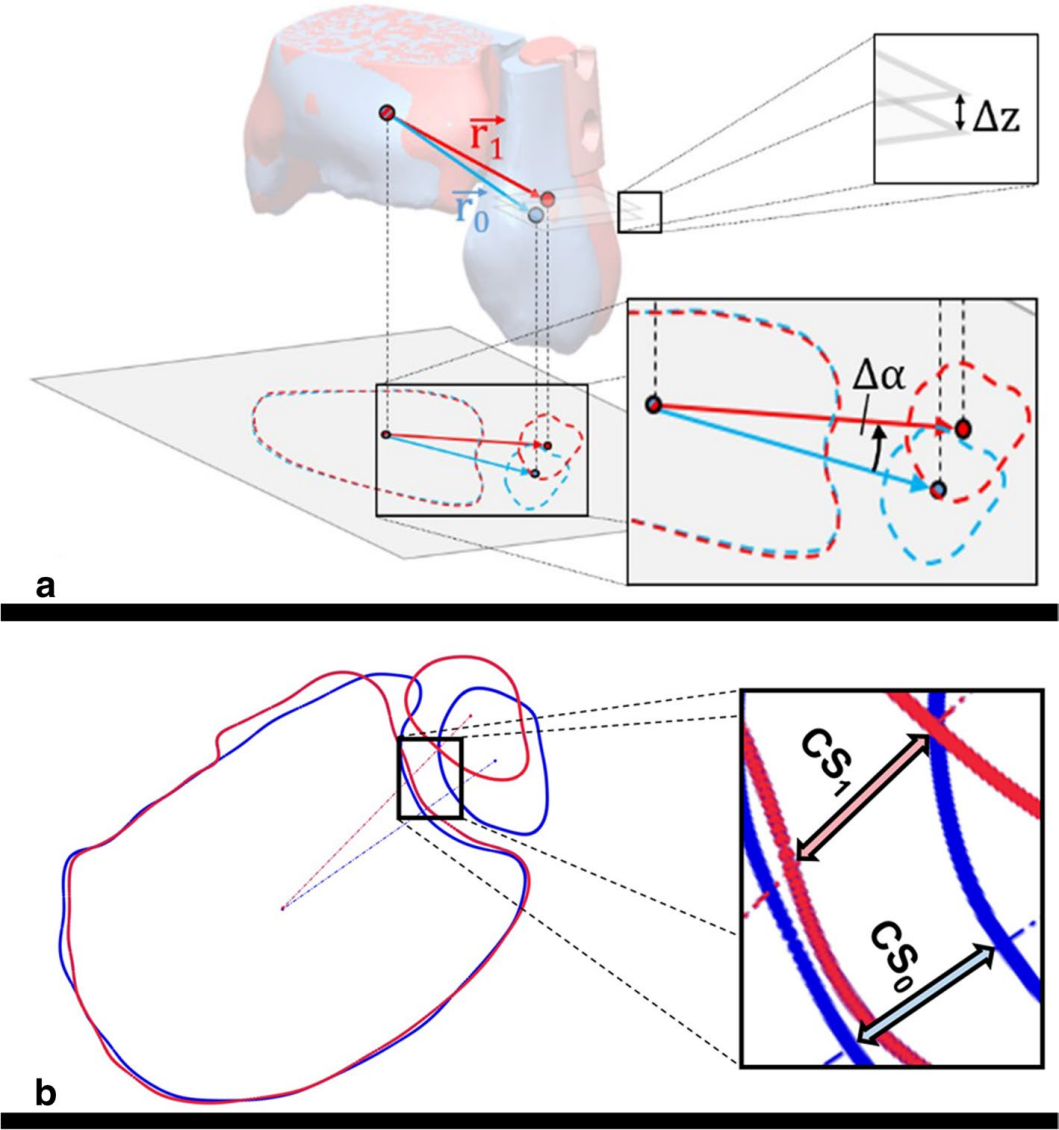
**a** Visualization of tibial and fibular centers of volume of native (blue) and operated (red) side, respective connection vectors ($\overrightarrow{r_0}$_,_ $\overrightarrow{r_1}$), and measurements of vertical offset (Δz) and translation angle (Δα) according to Souleiman et al. [14]. A case with dorsal malreduction of the fibula at the stabilized ankle (red) is illustrated. The osteosynthesis plate (red model) has been left in this model to illustrate the operated side. These were removed when calculating the parameters (see 2b). **b** Visualization of tibio-fibular clear space (*CS*_0_, *CS*_1_) measurement for native (blue) and operated (red) side (b)

**Fig. 2:**
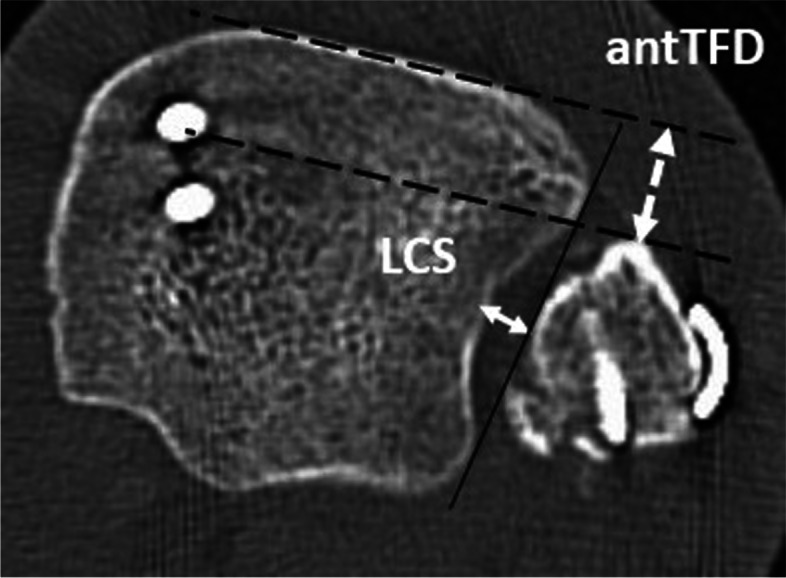
Computed tomography with transversal plain 10 mm proximal to the tibial plafond of a left angle after osteosynthesis of the lateral (two screws) and medial (plate and screws) malleolus to illustrate the 2D measurement of tibio-fibular clear space (LCS) and anterior tibio-fibular distance (antTFD)

All CT scans were performed within 3 days after surgery using a multidetector CT scanner (iCT 256, Philips, Best, The Netherlands). Patients were positioned supine and feet first with the ankle in neutral position. The scan area included bony structures at least 10 cm proximal to the distal tibial plateau. DICOM datasets with a slice thickness of ≤2 mm were further segmented using a dedicated 3D image processing software (Mimics 22.0, Materialise, Leuven, Belgium). Implants were virtually removed and resulting osseous defects were virtually filled considering the anatomical geometry. To specify the three-dimensional post-operative anatomy, 3D imaging-based computerized measurements including the tibio-fibular clear space (∆CS), translation angle (∆α) and vertical offset (∆z) were analyzed as described in a previous work (Fig. 1 a-b) [14]. ∆CS was defined as difference between injured and uninjured side, with positive ∆CS representing diastasis of the operative side. Positive ∆α implies dorsal translation of fibula in relation to tibia, negative angles representing anterior translation. Positive ∆z implies fibular shortening to the reference fibula. Syndesmotic malreduction was defined as ∆CS more than 2 mm, |∆α| more than 5° or |∆z| more than 3 mm, based on their absolute values [2, 7, 14]. For verification of 3D parameters, measurement of tibio-fibular clear space (LCS) according to Leporjärvi and anterior tibio-fibular distance (antTFD) according to Ahrberg was performed using axial CT images approximately 10 mm proximal to the tibial plafond of both sides (Fig. 2) [13, 29]. These parameters were selected due to their high intra-observer and inter-observer reliability in evaluating side differences, as demonstrated in previous studies [13, 30]. Again, the differences between injured and uninjured sides were calculated (ΔLCS, ΔantTFD). Positive LCS means diastasis of the syndesmosis and positive ΔantTFD means posterior translation of the fibula in relation to the tibia compared to the uninjured side, respectively.

### Statistics

Statistical analysis was performed with SPSS software (version 25, Chicago, IL, USA). The Student’s t-test or Mann-Whitney U-test were used to compare continuous variables between the study groups depending on normal distribution and study size (Shapiro-Wilk test). Categorical variables were compared using Pearson’s Chi-square test or Fisher’s exact test. *P*-values (*p*) < 0.05 was considered as statistically significant. Spearman-Rho correlation coefficients (ρ) were used for correlation analysis of the 2D and 3D parameters, |ρ| were interpreted as poor |*ρ*| < 0.1), moderate 0.1 < |*ρ*| < 0.5 and good |*ρ*| > 0.50 [31].

## Results

### Demographics and groups

Forty-seven of 184 patients met the inclusion criteria of bilateral postoperative CT with a slice thickness of ≤2 mm and a scan area of the tibia at least 10 cm proximal to the plafond. The mean age was 48.2 years (range 24 to 87; SD 18.0 year), men (*N* = 28, in mean 42.0 years, SD 13.3 year) were younger (*p* = 0.012) than women (*N* = 19, in mean 57.3 years, SD 20.5 year).

Eighteen patients were stabilized with SYS (SYS group) and 29 patients with SBS (SBS group). There were no significant differences in age or sex distribution between the groups (*p* = 0.661, *p* = 0. 866; Table 2).

**Table 2:** Patients’ demographic data, values of 2D and 3D parameters and statistical analyses

	SYS (*N* = 18)	SBS (*N* = 29)	*P* value (p)
Age [years] (SD; range)	50.3 (20.7; 24–87)	46.8 (16.4; 24–80)	0.661^a^
Sex female:male	7:11	12:17	0.866^b^
CS stabilized ankle [mm]	3.2 (1.6; 1.0–6.9)	3.6 (1.8; 0.1–9.1)	0.482^c^
CS uninjured ankle [mm]	2.9 (0.9; 1.6–4.6)	3.4 (1.1; 1.8–5.8))	0.098^c^
ΔCS [mm]	0.3 (1.1; −1.1-3.2)	0.2 (1.2; −1.9-3.4)	0.710^a^
LCS stabilized ankle [mm]	3.8 (1.5; 0.5–6.6)	3.8 (1.5; 0.8–7.6)	0.292^c^
LCS uninjured ankle [mm]	2.9 (1.1; 0.3–4.7)	3.6 (1.3; 0.6–6-0)	0.107^c^
ΔLCS [mm]	0.4 (1.4; −2.0-3.3)	0.3 (1.1; −1.4-2.1)	0.714^c^
Δα [°]	0.5 (4.6; −8.4-7.8)	2.1 (3.7; − 3.9-11.5)	0.192 ^c^
antTFD stabilized ankle [mm]	10.7 (3.9; 4.5–20.5)	12.2 (3.8; 3.2–19.9)	0.190^c^
antTFD uninjured ankle [mm]	11.1 (2.8; 6.4–18.0)	10.8 (3.2; 4.5–16.4)	0.744^c^
ΔantTFD [mm]	−0.4 (2.5; − 4.8-3.7)	1.4 (3.0; − 4.0-9.0)	0.037^c^
Δz [mm]	0.5 (2.4; −3.2-5.5)	0 (1.2; − 3.0-2.1)	0.477^a^

All data is presented as mean (SD; range). *CS* Clear space, *LCS* Leporjärvi clear space, *ΔLCS* Side-to-side difference of LCS, *Δα* Translational angle, *antTFD* Anterior tibiofibular distance; ΔantTFD = side-to-side difference of antTFD, Δz = vertical offset; ^a^Mann-Whitney-U-Test; ^b^Chi-Square test; ^c^Student’s *t*-test

### Statistical outcome of 3D parameters between groups

**Fig. 3:**
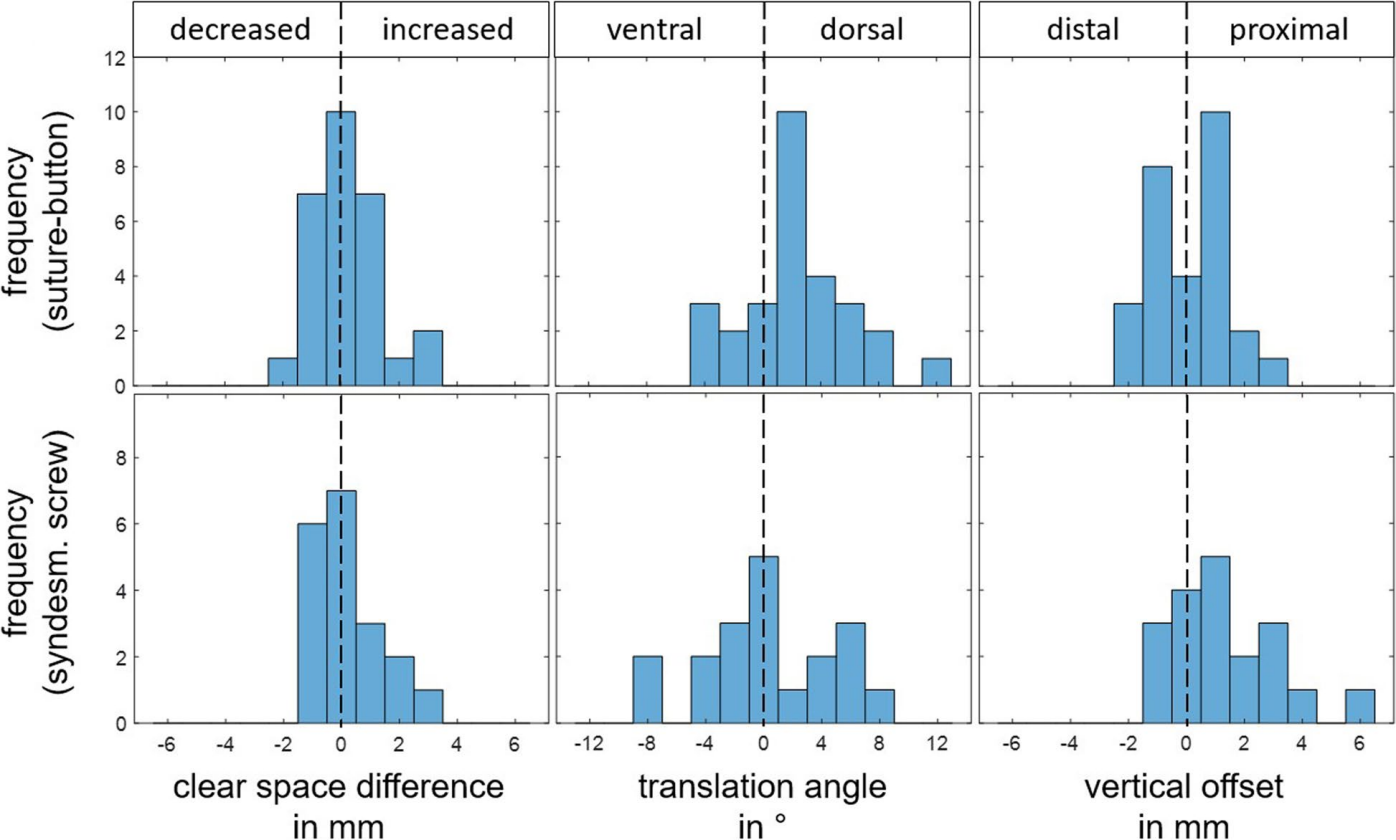
Histograms regarding tibio-fibular clear space difference, vertical offset and translation angle for patients stabilized with suture-button or syndesmotic screw with consideration of their direction

Regardless of the stabilization, the mean CS at the stabilized ankle was 3.5 mm (range 0.1 to 9.14 mm; SD 1.7 mm). At the stabilized ankle joints, a mild diastasis of ΔCS of 0.2 mm (range − 1.97 to 3.4 mm; SD 1.1 mm) was present in the side-to-side comparison. Both the SYS group, with a mean ΔCS of 0.3 mm (SD 1.1 mm), and the SBS group, with a mean ΔCS of 0.2 mm (SD 1.2 mm), had mild diastasis after stabilization in the side-to-side comparison, which did not differ between the two groups (*p* = 0.710, Table 2, Fig. 3). Further, both stabilization methods showed a slight dorsalization of the fibula to the tibia on the stabilized ankle, as reflected by a mean Δα of 0.5° in the SYS group (SD 4.6°) and a mean Δα of 2.0° in the SBS group (SD 3.7°, *p* = 0.192; Table 2, Fig. 3). Restoration of the fibula-to-tibia length ratio also did not differ between the two stabilization methods, with a mean Δz of 0.5 mm (SD 2.4 mm) for SYS and a mean Δz of 0 mm (SD 1.2 mm) for SBS (*p* = 0.363, Table 2, Fig. 3).

### Rating of reduction

Syndesmotic malreduction was found in 12 patients (26%) according to Δα, in five patients (11%) according to Δz and in three patients (6%) in assessment of ΔCS. One patient had a combination of ΔCS > 2 mm and Δz < − 3 mm and one patient of ΔCS > 2 mm and Δz > 3 mm. No combined Δα and ΔCS malreduction was detected (Table 3). Increased posterior translation was the most frequent reason for evaluation as syndesmotic malreduction in coronal and sagittal plane, with 66% (*N* = 10/15). The overall rate of syndesmotic malreduction in sagittal and coronal plane was 32% (*N* = 15/47). Seven patients (39%) of the SYS group and eight patients (28%) of the SBS group were assessed as malreduced (*p* = 0.255, Table 3).

**Table 3:** Rates of malreduction, statistical analysis between the groups

		SYS (*N* = 18)	SBS (*N* = 29)	*P*-value
ΔCS	anatomical	17	27	1.000^a^
	malreduction	1	2	
ΔLCS	anatomical	17	28	
	malreduction	1	1	
Δα	anatomical	12	23	0.234^b^
	post. Mal.	4 ^c^	6 ^c^	
	ant. Mal.	2 ^c^	0 ^c^	
ΔantTFD	anatomical	9	16	
	post. Mal.	3	11	
	ant. Mal.	6	2	
Δz	anatomical	13	29	0,006^b^
	shortening	3	0	
	lengthening	2	0	

*ΔCS* Side-to-side difference of tibiofibular clear space, Δα = translational angle; Δz = vertical offset; ^a^ Chi-Square test; ^b^ Fisher’s Exact Test

### Correlation of the 3D with 2D parameters

Mean ΔLCS was 0.3 mm (SD 1.2 mm) and mean ΔantTFD was 0.7 mm (SD 2.9 mm). The detailed absolute values of 3D and 2D measurements of the injured and uninjured sides are shown in Table 2. The Spearman-Rho correlation coefficients (ρ) between ΔCS and ΔLCS was 0.567 and 0.671 between Δα and ΔantTFD.

## Discussion

The aim of this study was to compare the quality of syndesmosis reduction and fixation performed with either SYS or SBS using standardized 3D-based measurement techniques.

The underlying null hypothesis that both procedures result in comparable immediate post-operative alignment of the syndesmosis assessed with 3D-based method could not be rejected. Both stabilization methods showed a similar rate of syndesmotic malreduction. Increased posterior translation was the most frequent reason for evaluation as syndesmotic malreduction. For the used measurements of the mediolateral and anteroposterior alignment, a good correlation of established 2D measurements and the 3D measurements could be shown.

Currently, there are few cadaver studies evaluating the outcome after stabilization of the syndesmosis using 3D-based measurement methods [30, 32]. In contrast to these, we found a mild diastasis in mean (0.2 mm) in both SYS and SBS stabilization. Also, our results confirmed that stabilization with SYS or SBS provided comparable results analyzed with 3D measurements [30]. In contrast to these 3D results, studies with two-dimensional measurements have reported a significant difference between SYS and SBS, with increased clear space in SYS in sense of diastasis of the syndesmosis [10, 23]. Clinical studies have shown that diastasis in side-to-side comparison of more than 2 mm is associated with a worse outcome [2, 7, 23]. Therefore, this value is used in numerous studies as a cut off value for evaluation as a malreduction [2, 9, 22, 33].

It has been shown that 2D measurements cannot fully describe the three-dimensional relationships of the syndesmosis [15, 16]. When considering the CS alone, the low rates of syndesmotic malreduction are confirmed by the 3D measurement. Comparable to our 3D analysis, 2D studies considering reduction in coronal and sagittal planes separately also showed that malreduction was more frequent in the sagittal plane with mostly posterior translation than in the coronal plane [10, 11]. Using a 3D comparison of the uninjured side with the side of a syndesmosis lesion within fractures, Burssens et al. show a dorsal translation of the fibula of − 4.7 mm in mean [15]. In addition, the sagittal alignment of the syndesmosis was independent of plantar or dorsiflexion [34]. These findings should be considered intra-operatively when reducing the fibula. The rate of syndesmotic malreduction independent of the surgical procedure was comparable to other studies [2, 7, 10, 23, 35]. In contrast to our results, lower rates of malreduction ranging from 0 to 16% have been reported for the SBS, with studies using inconsistent parameters [22, 23, 36, 37]. This is due to our separate consideration of coronary and sagittal malreductions on the one hand and exclusion of patients with inta-operative CT on the other. Based on intra-operative CT findings, the alignment of the fibula in the tibio-fibular incisura could be improved by immediate readjustment in 25.5% [38]. Therefore, in any treatment of syndesmotic injuries intra-operative three-dimensional imaging or at least post-operative computed tomography is recommended [38]. Based on these data, the clinic’s internal standard of open reduction was extended to include direct visualization of the syndesmosis and rigorous bilateral intra-operative 3D imaging in the treatment of syndesmotic injuries.

To our knowledge, this is the first 3D-based in vivo analysis of post-operative radiological outcome after stabilization of the syndesmotic injuries. The 3D measurements were independent of anatomical landmarks, allowing them to be carried out repetitively and independent of the examiner. Side-to-side differences can be measured exactly afterwards. Currently, no validated 3D measurement parameters are available, so the comparison of 3D parameters with two-dimensional CT can be rated as a limitation. However, the performed correlation of these 3D parameters with established 2D parameters showed a good correlation.

The main limitations of the used 3D technique are described in a previous work by Souleiman et al. [14]. Also, validation of the method on non-anatomically reduced fractures is still needed [14]. Previous studies on this topic used different parameters to assess the syndesmosis, making comparison difficult [9, 22, 23, 36]. It has been demonstrated that a side-to-side difference in the width of the syndesmosis of more than 2 mm is associated with a worse clinical outcome [2, 4, 5, 7]. At what level of malalignment revision surgery will result in a better outcome than non-revision is not known and should be the subject of further clinical investigation.

It might be discussed that non-weight-bearing CT scans were used for this study. But, the value of weight-bearing CT imaging for assessment of post-operative reduction is still controversial. Pre- and intra-operative CT scans are also unloaded [38–40]. In preoperative planning for reduction of syndesmotic lesions non-bearing cone-beam CT with 3D analysis, as performed in this or other studies, offers a low-radiation alternative to conventional CT [15, 32].

Also, the creation of 3D volumes and analysis of their configuration is still resource and time consuming. With further development of computer software, improved imaging, and its automated processing, it is expected that this limitation will decrease in the future. This will enable a clinical evaluation and classification of these 3D measurements as well as reevaluation of the method on a larger number of cases. Which stabilization procedure should be preferred is still being debated [20, 21, 41–44]. Recent studies show a better functional outcome after SBS stabilization [17, 21, 41, 45]. It remains to be seen whether analysis of the relationship between the 3D and clinical parameters will reveal differences between the two stabilization techniques and identify causative factors, if appropriate.

## Conclusion

The analysis of three-dimensional volume bodies has shown no differences in immediate post-operative alignment of the syndesmosis with either syndesmotic screw or suture-button system. Comparable rates of syndesmotic malreduction after open stabilization could be shown. A separate evaluation of the congruency of the syndesmosis in each plane is recommendable. Special attention should be paid to syndesmotic malreduction in the sagittal orientation of the fibula in relation to the tibia in radiological control of the syndesmotic congruity as well as intra-operatively.

## Data Availability

The datasets used and/or analyzed during the current study are available from the corresponding author on reasonable request.

## Abbreviations

2D: Two-dimensional
3D: Three-dimensional
∆α: Translation angle
∆CS: Tibio-fibular clear space and vertical offset
∆z: Vertical offset
Ρ: Spearman-Rho correlation coefficients
antTFD: Anterior tibio-fibular distance
CT: Computed tomography
DICOM: Digital Imaging and Communications in Medicine
LCS: Leporjärvi clear space
SBS: Suture button system
SD: Standard deviation
SYS: Syndesmosis screw

## References

[1] Rüedi TP, Murphy WM (2000). AO principles of fracture management.

[2] Andersen MR, Diep LM, Frihagen F, Castberg Hellund J, Madsen JE, Figved W (2019). Importance of Syndesmotic reduction on clinical outcome after Syndesmosis injuries. J Orthop Trauma.

[3] Litrenta J, Saper D, Tornetta P, Phieffer L, Jones CB, Mullis BH (2015). Does Syndesmotic injury have a negative effect on functional outcome? A multicenter prospective evaluation. J Orthop Trauma.

[4] Ray R, Koohnejad N, Clement ND, Keenan GF (2019). Ankle fractures with syndesmotic stabilisation are associated with a high rate of secondary osteoarthritis. Foot Ankle Surg.

[5] van Vlijmen N, Denk K, van Kampen A, Jaarsma RL (2015). Long-term results after ankle Syndesmosis injuries. Orthopedics..

[6] Hunt KJ, Goeb Y, Behn AW, Criswell B, Chou L (2015). Ankle joint contact loads and displacement with progressive Syndesmotic injury. Foot Ankle Int.

[7] Sagi HC, Shah AR, Sanders RW (2012). The functional consequence of Syndesmotic joint Malreduction at a minimum 2-year follow-up. J Orthop Trauma.

[8] Beumer A, van Hemert WLW, Niesing R, Entius CAC, Ginai AZ, Mulder PGH (2004). Radiographic measurement of the distal Tibiofibular Syndesmosis has limited use. Clin Orthop Relat Res.

[9] Gardner MJ, Demetrakopoulos D, Briggs SM, Helfet DL, Lorich DG (2006). Malreduction of the Tibiofibular Syndesmosis in ankle fractures. Foot Ankle Int..

[10] Davidovitch RI, Weil Y, Karia R, Forman J, Looze C, Liebergall M (2013). Intraoperative Syndesmotic reduction: three-dimensional versus standard fluoroscopic imaging. J Bone Joint Surg.

[11] Boszczyk A, Kwapisz S, Krümmel M, Grass R, Rammelt S (2019). Anatomy of the tibial incisura as a risk factor for syndesmotic injury. Foot Ankle Surg.

[12] Bartonicek J (2003). Anatomy of the tibiofibular syndesmosis and its clinical relevance. Surg Radiol Anat.

[13] Ahrberg AB, Hennings R, von Dercks N, Hepp P, Josten C, Spiegl UJ (2020). Validation of a new method for evaluation of syndesmotic injuries of the ankle. Int Orthop (SICOT).

[14] Souleiman F, Heilemann M, Hennings R, Hennings M, Klengel A, Hepp P (2021). A standardized approach for exact CT-based three-dimensional position analysis in the distal tibiofibular joint. BMC Med Imaging.

[15] Burssens A, Vermue H, Barg A, Krähenbühl N, Victor J, Buedts K (2018). Templating of Syndesmotic ankle lesions by use of 3D analysis in Weightbearing and Nonweightbearing CT. Foot Ankle Int..

[16] Barg A, Bailey T, Richter M, de Cesar NC, Lintz F, Burssens A (2018). Weightbearing computed tomography of the foot and ankle: emerging technology topical review. Foot Ankle Int..

[17] Shimozono Y, Hurley ET, Myerson CL, Murawski CD, Kennedy JG (2019). Suture button versus Syndesmotic screw for Syndesmosis injuries: a Meta-analysis of randomized controlled trials. Am J Sports Med.

[18] Zhang P, Liang Y, He J, Fang Y, Chen P, Wang J (2017). A systematic review of suture-button versus syndesmotic screw in the treatment of distal tibiofibular syndesmosis injury. BMC Musculoskelet Disord.

[19] Onggo JR, Nambiar M, Phan K, Hickey B, Ambikaipalan A, Hau R (2020). Suture button versus syndesmosis screw constructs for acute ankle diastasis injuries: a meta-analysis and systematic review of randomised controlled trials. Foot Ankle Surg.

[20] Laflamme M, Belzile EL, Bédard L, van den Bekerom MPJ, Glazebrook M, Pelet S (2015). A prospective randomized multicenter trial comparing clinical outcomes of patients treated surgically with a static or dynamic implant for acute ankle Syndesmosis rupture. J Orthop Trauma.

[21] Andersen MR, Frihagen F, Hellund JC, Madsen JE, Figved W (2018). Randomized trial comparing suture button with single Syndesmotic screw for Syndesmosis injury. J Bone Joint Surg.

[22] Kortekangas T, Savola O, Flinkkilä T, Lepojärvi S, Nortunen S, Ohtonen P (2015). A prospective randomised study comparing TightRope and syndesmotic screw fixation for accuracy and maintenance of syndesmotic reduction assessed with bilateral computed tomography. Injury..

[23] Naqvi GA, Cunningham P, Lynch B, Galvin R, Awan N (2012). Fixation of ankle Syndesmotic injuries: comparison of TightRope fixation and Syndesmotic screw fixation for accuracy of Syndesmotic reduction. Am J Sports Med.

[24] Beerekamp MSH, de Muinck Keizer RJO, Schepers T, Beenen LFM, Luitse JSK, Schep NW (2019). The correlation between intra-operative 2D- and 3D fluoroscopy with postoperative CT-scans in the treatment of calcaneal fractures. Eur J Radiol.

[25] Bai J, Wang Y, Zhang P, Liu M, Wang P, Wang J (2018). Efficacy and safety of 3D print-assisted surgery for the treatment of pilon fractures: a meta-analysis of randomized controlled trials. J Orthop Surg Res.

[26] Meinberg E, Agel J, Roberts C, Karam M, Kellam J (2018). Fracture and dislocation classification compendium—2018. J Orthop Trauma.

[27] Buckley RE, Moran CG, Apivatthakakul T. AO principles of fracture management volume 1. Volume. 2017;1.

[28] Pakarinen H, Flinkkilä T, Ohtonen P, Hyvönen P, Lakovaara M, Leppilahti J (2011). Intraoperative assessment of the stability of the distal Tibiofibular joint in supination-external rotation injuries of the ankle: sensitivity, specificity, and reliability of two clinical tests. J Bone Joint Surg.

[29] Lepojärvi S, Pakarinen H, Savola O, Haapea M, Sequeiros RB, Niinimäki J (2014). Posterior translation of the fibula may indicate Malreduction: CT study of Normal variation in uninjured ankles. J Orthop Trauma.

[30] Schon JM, Williams BT, Venderley MB, Backus JD, Dornan GJ, Turnbull TL (2016). 3-D CT analysis of screw and suture-button fixation for Syndesmosis repair. Foot Ankle Orthop.

[31] Cohen J (1988). Statistical power analysis for the behavioral sciences.

[32] Ebinger T, Goetz J, Dolan L, Phisitkul P (2013). 3D model analysis of existing CT syndesmosis measurements. Iowa Orthop J.

[33] Thordarson DB, Motamed S, Hedman T, Ebramzadeh E, Bakshian S (1997). The effect of fibular Malreduction on contact pressures in an ankle fracture Malunion model*: the journal of Bone & Joint. Surgery..

[34] Wong MT, Wiens C, Lamothe J, Edwards WB, Schneider PS. Four-dimensional CT analysis of Normal Syndesmotic motion. Foot Ankle Int. 2021;107110072110152. 10.1177/10711007211015204 PMC8592109 34088231

[35] Mukhopadhyay S, Metcalfe A, Guha AR, Mohanty K, Hemmadi S, Lyons K (2011). Malreduction of syndesmosis—are we considering the anatomical variation?. Injury..

[36] Miller AN, Carroll EA, Parker RJ, Boraiah S, Helfet DL, Lorich DG (2009). Direct visualization for Syndesmotic stabilization of ankle fractures. Foot Ankle Int..

[37] Sanders D, Schneider P, Taylor M, Tieszer C, Lawendy A-R, Society COT (2019). Improved reduction of the Tibiofibular Syndesmosis with TightRope compared with screw fixation: results of a randomized controlled study. J Orthop Trauma.

[38] Franke J, von Recum J, Suda AJ, Grützner P, Wendl K, Bone J (2012). Intraoperative three-dimensional imaging in the treatment of acute unstable syndesmotic injuries. TIJD Traumatologie.

[39] Hamard M, Neroladaki A, Bagetakos I, Dubois-Ferrière V, Montet X, Boudabbous S (2020). Accuracy of cone-beam computed tomography for syndesmosis injury diagnosis compared to conventional computed tomography. Foot Ankle Surg.

[40] Kumar V, Baburaj V, Patel S, Sharma S, Vaishya R (2021). Does the use of intraoperative CT scan improve outcomes in Orthopaedic surgery? A systematic review and meta-analysis of 871 cases. J Clin Orthop Trauma.

[41] Ræder BW, Figved W, Madsen JE, Frihagen F, Jacobsen SB, Andersen MR (2020). Better outcome for suture button compared with single syndesmotic screw for syndesmosis injury: five-year results of a randomized controlled trial. Bone Joint J.

[42] Naqvi GA, Shafqat A, Awan N (2012). Tightrope fixation of ankle syndesmosis injuries: clinical outcome, complications and technique modification. Injury..

[43] Kocadal O, Yucel M, Pepe M, Aksahin E, Aktekin CN (2016). Evaluation of reduction accuracy of suture-button and screw fixation techniques for Syndesmotic injuries. Foot Ankle Int..

[44] Kim J-H, Gwak H-C, Lee C-R, Choo H-J, Kim J-G, Kim D-Y (2016). A comparison of screw fixation and suture-button fixation in a Syndesmosis injury in an ankle fracture. J Foot Ankle Surg.

[45] McKenzie AC, Hesselholt KE, Larsen MS, Schmal H (2019). A systematic review and Meta-analysis on treatment of ankle fractures with Syndesmotic rupture: suture-button fixation versus cortical screw fixation. J Foot Ankle Surg.

